# Community detection in multi-frequency EEG networks

**DOI:** 10.1038/s41598-023-35232-2

**Published:** 2023-05-19

**Authors:** Abdullah Karaaslanli, Meiby Ortiz-Bouza, Tamanna T. K. Munia, Selin Aviyente

**Affiliations:** Department of Electrical and Computer Engineering, Michigan State University, East Lansing, MI 48824 USA

**Keywords:** Complex networks, Mathematics and computing

## Abstract

Functional connectivity networks of the human brain are commonly studied using tools from complex network theory. Existing methods focus on functional connectivity within a single frequency band. However, it is well-known that higher order brain functions rely on the integration of information across oscillations at different frequencies. Therefore, there is a need to study these cross-frequency interactions. In this paper, we use multilayer networks to model functional connectivity across multiple frequencies, where each layer corresponds to a different frequency band. We then introduce the multilayer modularity metric to develop a multilayer community detection algorithm. The proposed approach is applied to electroencephalogram (EEG) data collected during a study of error monitoring in the human brain. The differences between the community structures within and across different frequency bands for two response types, i.e. error and correct, are studied. The results indicate that following an error response, the brain organizes itself to form communities across frequencies, in particular between theta and gamma bands while a similar cross-frequency community formation is not observed following the correct response.

## Introduction

Advances in neuroimaging technologies allow the brain to be modeled as a complex network, where the nodes correspond to the different brain units and the edges represent structural or functional connections among the units^1^. In order to characterize the topology and dynamics of brain networks, various descriptive and inferential network measures such as centrality, degree distribution and small-worldness^2–6^ with respect to disease, task, learning, cognitive control, attention and memory^1,4,6,7–11^ are utilized. Current network models have been mostly limited to examining a single network instance either of a subject, a frequency band or a task. However, most neurophysiological recordings, such as the electroencephalogram (EEG), allows one to capture brain dynamics across multiple temporal and spatial scales. Reducing this rich information into a single network disregards the high amount of dependency that exists between networks of different subjects, frequency bands or tasks. Thus, a principled mathematical framework to accurately study this multiplicity of brain connectivity is needed.

**Figure 1:**
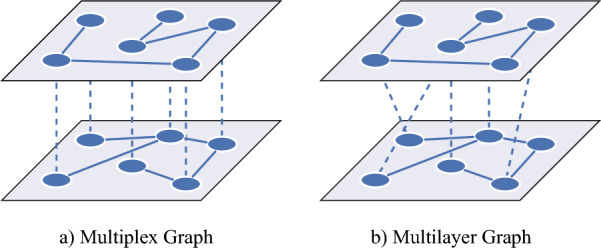
Illustration of two-layer multiplex and multilayer graphs: (**a**) Multiplex graph, where inter-layer edges are allowed only between nodes representing the same physical entities. (**b**) Multilayer graph, where inter-layer edges are allowed between any two nodes corresponding to different physical entities.

Recently, multilayer networks^12–14^ have been proposed as a mathematical framework to study multiple networks simultaneously. Multilayer networks consist of multiple layers, each of which carry information from a different network while inter-layer edges represent the dependency between these networks. Due to their ability to represent and study multi-dimensional and multi-scale data, multilayer networks have gained attention in network neuroscience^15–20^. Initial work to model multiplicity of brain connectivity primarily employs multiplex networks (Fig. 1a), which are restricted versions of multilayer networks where inter-layer edges are only allowed between nodes corresponding to the same brain regions. The meaning of layer in these multiplex brain networks can vary depending on context, such as different modalities, subjects, and frequency bands. For example, Battiston et al.^21^ introduce a two layer network combining structural and functional modalities using diffusion tensor imaging (DTI) and functional MRI (fMRI), respectively. Another line of work considers multiplex networks, where each layer corresponds to a different subject, to investigate intra- and inter-subject variability of brain connectivity^22^. Another example is the temporal or dynamic network where each layer represents the interplay between brain regions over some time window^5,7,23^. The inter-layer edges between time windows are added only between a node and itself in an adjacent time window. This approach has been used to analyze the temporal evolution of network modules and examine dynamic reconfiguration and “flexibility” of functional networks. Finally, multiplex networks where each layer corresponds to the connectivity in different frequency bands are considered to study the connectivity across multiple frequency bands, simultaneously^24–28^. While this line of work reveals important characteristics of multiplicity of brain connectivity, as aforementioned it restricts inter-layer edges by using multiplex networks. Recently, this restriction on inter-layer edges has been removed by modeling the brain connectivity using multilayer networks, where inter-layer edges are allowed between any brain regions (Fig. 1b)^17,29–31^. For example, magnetoencephalography (MEG^30,31^) and EEG^17,32^ recordings are used to construct functional multilayer networks, where each layer corresponds to the links within a frequency band, and the inter-layer edges correspond to the cross-frequency coupling across frequency bands.

Topological characteristics of multiplex and multilayer brain networks have been analyzed with various graph theoretical tools, such as hub node identification^16^, motif analysis^21^ and algebraic connectivity^31^. An important tool in the analysis of graphs is community detection^33^. Communities are defined as groups of nodes that are more strongly connected among themselves than they are to the rest of the network. Various community detection methods have been developed and applied to single-layer brain networks to find communities, which often correspond to specialized functional subnetworks of the brain^34,35^. Although these methods can be applied to multiplex and multilayer graphs, they do not achieve good performance as they do not take the heterogeneity of connections across layers. Thus, recent work aims to extend community detection methods to these high-dimensional graphs^36–40^. However, most of these extensions are limited to multiplex networks except the following recent work. Pramanik *et al.*^39^ extends the definition of modularity to multilayer networks. The proposed multilayer modularity metric is maximized using Girvan-Newman and Louvain algorithms. However, this approach does not take the resolution limit of modularity into account^39^, limiting its practical use. Chen *et al.*^40^, on the other hand, extends the definition of normalized cut to multilayer networks by constructing a block supra-Laplacian matrix and proposes a spectral clustering algorithm based on this supra-Laplacian matrix. Although the method is developed for multilayer networks, it does not take the heterogeneity of inter-layer edge weights into account.

**Figure 2:**
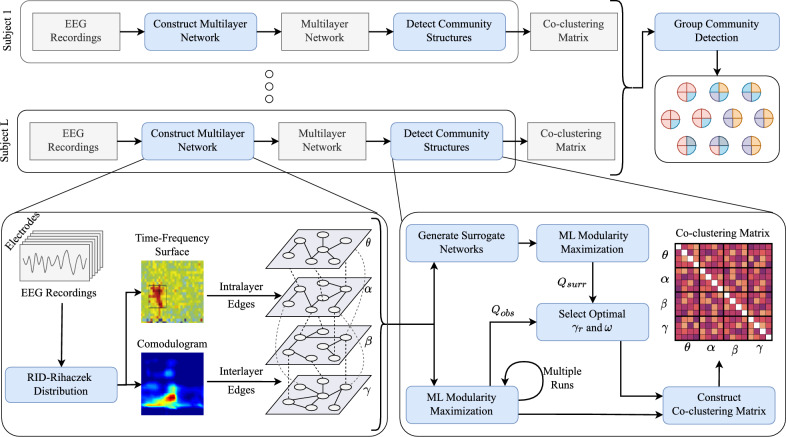
Flowchart of the proposed approach for community detection of multi-frequency EEG networks. Bottom two panels illustrate multilayer network construction (left) and community detection for each subject (right).

In this paper, we aim to characterize the topological organization of multilayer brain networks through multilayer community detection. In order to achieve this goal, we first construct multi-frequency networks from EEG data, where the intra- and inter-layer edges are quantified by previously published time-frequency phase synchrony^41^ and phase amplitude coupling (PAC)^42^ measures, respectively. Thus, the constructed network is a multilayer network with inter-layer edges allowed between all brain regions. Next, a new multilayer modularity metric is defined based on a multilayer null model that preserves the layer-wise node strengths while randomizing the remaining characteristics of the network. The proposed modularity is parameterized with resolution parameter to handle the resolution limit of modularity, and inter-layer scale parameter to control the importance of inter-layer edges in community formation. The optimal values of these parameters are determined using a surrogate data based procedure. Third, a group community detection method is proposed to find the common community structure for a set of subjects. The method uses subjects’ co-clustering matrices obtained from multiple runs of modularity maximization, thus it is able to address the issue of degeneracy in modularity maximization^43^. Finally, the group level differences between the two response types during Flanker task, i.e., error and correct, are evaluated from a multi-frequency network perspective. The proposed approach is outlined in Fig. 2.

## Materials and methods

### EEG data

The EEG data was acquired during a cognitive control-related error processing task where the subjects performed a letter version of the speeded reaction Flanker task^44^. The experimental protocol of this study was approved by the Institutional Review Board (IRB) of the Michigan State University (IRB: LEGACY13-144). The data collection was conducted by following the regulations approved by this protocol. Prior to data acquisition, all subjects signed an informed and written consent form. The EEG signals were recorded with a BioSemi ActiveTwo system using a cap with 64 Ag-AgCl electrodes placed at standard locations of the International 10-20 system. The sampling rate of the data was 512 Hz. After using standard artifact rejection algorithms^45^, volume conduction was minimized using the Current Source Density (CSD) Toolbox^46^.

During recording, each subject was presented with a string of five letters at each trial. Letters could be congruent (e.g., SSSSS) or incongruent stimuli (e.g., SSTSS) and the subject was instructed to respond to the center letter with a standard mouse. The trials started with a flanking stimulus (e.g., SS SS) of 35 ms followed by the target stimuli (e.g., SSSSS/SSTSS) displayed for about 100 ms. The total display time is 135 ms, followed by a 1200 to 1700 ms inter-trial break between the trials. These trials capture the Error-Related Negativity (ERN) after an error response and the Correct-Related Negativity (CRN) after a correct response. For each subject, 480 total trials (each of 1-second in duration) were recorded, where the number of error trials varied from 20 to 61 across the subjects. For a fair comparison between ERN/CRN, the same number of correct trials were selected randomly. As earlier studies suggested a rise in synchronization related to ERN for the 25–75 ms time window^47^, all of the analysis in this paper was conducted for the 25–75 ms time period following the response. For each subject and each response type (error and correct), a multilayer network with four layers is constructed where layers correspond to the four EEG frequency bands: $\theta$ (4–7 Hz), $\alpha$ (8–12 Hz), $\beta$ (13–30 Hz), $\gamma$ (31–100 Hz). In this paper, we consider data from 20 participants.

### Construction of multilayer EEG networks

#### Multilayer networks

An undirected multilayer network^12^ is a quadruplet $\mathscr {M}= (\mathscr {V}, \mathscr {L}, V, E)$ where $\mathscr {V}$ is the set of physical entities, $\mathscr {L}$ is the set of layers with $|\mathscr {L}| = L$. $V \subseteq \mathscr {V}\times \mathscr {L}$ with $|V|=N$ is the set of nodes, which are representations of physical entities in layers and $E \subseteq V\times V$ is the edge set. Nodes are represented as ${u^{h}}$, where $u \in \mathscr {V}$ and ${{h}\in \mathscr {L}}$. An edge between ${u^{h}}$ and ${v^{k}}$ is denoted by ${e_{uv}^{{h}{k}}}$ and associated with the weight ${w_{uv}^{{h}{k}}}$. *V* can be partitioned into layers, i.e., ${V=\bigcup _{{h}=1}^L V^{h}}$ where ${V^{h}}$ is the set of nodes in layer ${{h}}$ with ${|V^{{h}}| = N^{{h}}}$. Similarly, *E* can be partitioned as ${E = \bigcup _{{h}=1}^L E^{{h}}\ \cup \ \bigcup _{{h}\ne {k}=1}^L E^{{h}{k}}}$, where ${E^{h}}$ is the set of intra-layer edges for layer ${{h}}$ and ${E^{{h}{k}}}$ is the set of inter-layer edges between nodes in layers ${{h}}$ and ${{k}}$. Using this notation, one can define intra-layer graphs ${G^{{h}}=(V^{{h}}, E^{h})}$ and bipartite inter-layer graphs ${G^{{h}{k}} = (V^{{h}}, V^{{k}}, E^{{h}{k}})}$. $\mathscr {M}$ can be represented by a

supra-adjacency matrix $\textbf{A}\in \mathbb {R}^{N\times N}$, defined as:

1 $$\begin{aligned} \textbf{A}= \begin{bmatrix} \textbf{A}^{1} &{} \quad \textbf{A}^{12} &{}\quad \dots &{} \quad \textbf{A}^{1L} \\ \textbf{A}^{21} &{} \quad \textbf{A}^{2} &{}\quad \dots &{} \quad \textbf{A}^{2L} \\ \vdots &{}\quad \vdots &{}\quad \ddots &{}\quad \vdots \\ \textbf{A}^{L1} &{}\quad \textbf{A}^{L2} &{} \quad \dots &{} \quad \textbf{A}^{L} \end{bmatrix}, \end{aligned}$$

where ${\textbf{A}^{{h}}}$ is the adjacency matrix of ${G^{h}}$ and ${\textbf{A}^{{h}{k}}}$ is the incidence matrix of the bipartite graph, ${G^{{h}{k}}}$. Layer-wise strength of a node ${u^{h}}$ is the sum of weights corresponding to the edges connected to the nodes in layer ${{k}}$, i.e., ${s_{u^{h}}^{k}= \sum _{v\in V^{{k}}} w_{uv}^{{h}{k}}}$ with ${w_{uv}^{{h}{k}} = 0}$ if ${e_{uv}^{{h}{k}} \not \in E^{{h}{k}}}$ (${E^{{h}}}$ if ${{h}= {k}}$).

#### Intra-layer edges

For a multilayer brain network where each layer corresponds to a different frequency band, the intra-layer edges correspond to functional connectivity and can be quantified using measures of correlation, coherence or phase synchrony. In prior work, we have illustrated the superior performance of reduced interference Rihaczek (RID-Rihaczek) time-frequency distribution-based phase synchrony index, i.e. RID-TFPS, in terms of time and frequency resolution and robustness to noise^41,48^. This complex time-frequency distribution can be utilized to calculate the phase difference $\phi _{u,v}(t,f)$, between two signals $x_u$ and $x_v$ as:

2 $$\begin{aligned} \phi _{u,v}(t,f) = \arg \bigg [\frac{C_u(t,f) C_{v}^*(t,f)}{| C_u(t,f) | | C_v(t,f) |}\bigg ], \end{aligned}$$

where $C_{u}(t,f)$ and $C_{v}(t,f)$ are the complex time-frequency distributions of $x_{u}$ and $x_{v}$, respectively. Phase Locking Value (PLV) quantifies the consistency of the phase differences across trials and is computed as follows^49^:

3 $$\begin{aligned} \text {PLV}_{u,v}(t,f) = \frac{1}{K}{\bigg | \sum _{k=1}^{K} e^{j\phi _{u,v}^k(t,f)}\bigg | }, \end{aligned}$$

where *K* is the total number of trials and $\phi _{u,v}^{k}(t,f)$ is the phase difference between $x_u^{k}$ and $x_v^{k}$ for trial *k*. After the pairwise PLV values are computed, the average pairwise synchrony within a predefined time window of interest, $W = [t_{1},t_{2}]$, and a chosen frequency band is used as intra-layer edge weights, i.e., ${w_{uv}^{{h}{h}}=\frac{1}{|W|}\frac{1}{|{h}|}\sum _{t \in W}\sum _{f \in {h}} \text {PLV}_{u,v} (t,f)}$, $1\le u,v \le N$, where *N* is the number of brain regions, |*W*| is the length of the time interval and ${|{h}|}$ is the bandwidth of the particular frequency band ${{h}}$.

#### Inter-layer edges

In a multilayer network, where the different layers correspond to different frequencies, the inter-layer edges can be quantified through measures of cross-frequency coupling. In particular, phase amplitude coupling (PAC) which computes the modulation of the amplitude/power of a high frequency rhythm by the phase of a slower frequency rhythm is a commonly used metric^50,51^. In prior work, we introduced a RID-Rihaczek time-frequency-based PAC measure and illustrated its superior performance with respect to Hilbert transform and wavelet-based methods^42,52^. To quantify PAC, we first extract the instantaneous amplitude envelope of the high frequency component at node *u*, $a^{u}_{f_a}(t)$, and the instantaneous low frequency phase component at node *v*, $\phi _{f_p}^{v}(t)$, using RID-Rihaczek distribution, where $f_{p}$ and $f_{a}$ are frequencies within the ${{h}}$th and ${{k}}$th frequency bands, respectively. $a^{u}_{f_a}(t)$ is obtained from the frequency constrained time marginal of $C_u(t,f)$ as:

4 $$\begin{aligned} a^{u}_{f_a}(t) = \int \nolimits _{f_{a_1}}^{f_{a_2}} {C_{u}(t,f)df}, \end{aligned}$$

where $f_{a_1}$ and $f_{a_2}$ is the bandwidth around the chosen high frequency. Similarly, the low frequency phase at node *v* is obtained from $C_v(t,f)$, as:

5 $$\begin{aligned} \phi ^{v}_{f_p}(t) = \arg \left[ \frac{C_{v}(t,{f_p})}{| C_{v}(t,{f_p}) |}\right] . \end{aligned}$$

Once the amplitude and phase components are extracted, PAC is estimated by distributing $a^{u}_{f_a}(t)$ and $\phi ^{v}_{f_p}(t)$ to a composite vector in the complex plane at each time point and measuring the direct PAC (dPAC)^53^:

6 $$\begin{aligned} \text {dPAC}_{{u,v}}(f_{p},f_{a},t) = \frac{1}{\sqrt{K}} \frac{\bigg | {\sum _{k=1}^{K}{a^{u,k}_{f_a}(t)}{e^{j\phi ^{v,k}_{f_p}(t)}}} \bigg |}{\sqrt{ {\sum _{k=1}^{K}{a^{u,k}_{f_a}(t)}^2}}}. \end{aligned}$$

This metric is selected to ensure that the intra- and inter-layer edges are both normalized and within the same range. The weights of the inter-layer edges between node *u* and *v* are computed as ${w_{uv}^{{h}{k}} = \frac{1}{|W|}\frac{1}{|{h}||{k}|}\sum _{t \in W}\sum _{f_{p}\in {h}}\sum _{f_{a}\in {k}}dPAC_{u,v}(f_{p},f_{a},t)}$.

### Multilayer modularity

Modularity function quantifies the quality of a partition by comparing the intra-community edge density to that expected under a null model and is calculated as follows^54^:

7 $$\begin{aligned} Q = \sum _{i=1}^N \sum _{j=1}^N (A_{ij} - \gamma _{r} P_{ij}) \delta _{g_ig_j}, \end{aligned}$$

where $P_{ij}$ is the expected edge weight between nodes *i* and *j* under the null model, $g_i$ is the community of node *i*, and $\delta _{g_{i}g_{j}} = 1$ if $g_i=g_j$ and 0, otherwise. $\gamma _{r}$ is the resolution parameter^55^ to overcome the resolution limit of modularity^56^. By tuning $\gamma _{r}$, one can change the resolution of the modularity function such that larger $\gamma _{r}$ values can detect smaller communities. The selection of $P_{ij}$ depends on the null model which is a random graph with some properties, *e.g.* edge density, of the observed network preserved. Different null models can be used to define $P_{ij}$ depending on the graph under study. For example in the configuration null model, the degree of each node is the same as that of the observed network so that the identified community structure is not affected by the heterogeneity of the degree distribution. This assumption is based on the fact that nodes with a high degree tend to connect with each other merely because they have high number of connections and not necessarily because they are within the same community^57^. To prevent this tendency to bias community detection, the null model preserves the node degrees. On the other hand, Erdős-Rényi null model does not make such an assumption and allows the identified community structure to be influenced by the degree distribution.

Based on this insight on the role of null models, we extend the definition of modularity function to multilayer networks by considering which properties of the observed multilayer network we want to preserve in the null model. In neuronal networks such as the multi-frequency brain networks, the edge weights are expected to be heterogeneous across layers^28,31^. This is due to the fact that after a given task, usually oscillations across only a subset of frequencies are activated. Thus, the edge weights across layers cannot be homogeneous. It is important to take this heterogeneity into account to prevent trivial partitions based on the layer label rather than the true community membership. Therefore, the null model used in the definition of the modularity function should preserve the heterogeneity of edge weights across layers. We define *multilayer configuration null model*, which preserves layer-wise node strengths while randomizing the remaining characteristics of the observed multilayer graph. The expected edge weight between ${u^{h}}$ and ${v^{k}}$ based on multilayer configuration null model is then defined as:

8 $$\begin{aligned} {P_{uv}^{{h}{k}} = \frac{s_{u^{h}}^{k}s_{v^{k}}^{h}}{(1+\delta _{{h}{k}}) m^{{h}{k}}},} \end{aligned}$$

where ${m^{{h}{h}}}$ is the total weight of the intra-layer edges in layer ${{h}}$, ${m^{{h}{k}}}$ is the total weight of the inter-layer edges between layers ${{h}}$ and ${{k}}$, and ${\delta _{{h}{k}} = 1}$ if ${{h}={k}}$ and 0, otherwise. The multilayer modularity is then defined as follows:

9 $$\begin{aligned} {Q = \sum _{{h}=1}^L \sum _{i=1}^{n^{h}} \sum _{j=1}^{n^{h}} (A_{ij}^{{h}{h}} - \gamma _{r} P_{ij}^{{h}{h}}) \delta _{g_i^{h}g_j^{h}} + \omega \sum _{{h}=1}^L \sum _{{k}=1}^L \sum _{i=1}^{n^{h}} \sum _{j=1}^{n^{k}} (A_{ij}^{{h}{k}} - \gamma _{r} P_{ij}^{{h}{k}}) \delta _{g_i^{h}g_j^{k}},} \end{aligned}$$

where $\gamma _{r}$ is the resolution parameter and $\omega$ is the scaling parameter that weighs the importance of inter-layer connections. Equation (9) can be optimized with greedy algorithms, such as the Louvain algorithm^58^, developed for maximizing the single-layer modularity function defined in (7). In this work, we use the Leiden algorithm, which is an extension of the Louvain algorithm with better performance^59^.

### Resolution parameter and inter-layer scale selection

We propose a statistical testing approach comparing the modularity value of the observed multilayer network to that of surrogate networks to determine the resolution and inter-layer scale parameters in (9). Since the multilayer EEG networks are fully connected and weighted, we focus on randomization techniques presented in^60^ and extend it for generating multilayer surrogate networks. In particular, we select two edges ${e_{uv}^{{h}{k}}}$ and ${e_{st}^{{l}{m}}}$ and swap their edge weights. Edges are selected such that ${{h}= {l}}$ and ${{k}= {m}}$, which ensures that the heterogeneity of edge weights across layers is preserved in the surrogate network.

Assume that we are given an observed multilayer network $\mathscr {M}$ and *c* surrogate multilayer networks generated from $\mathscr {M}$ as described above. We perform community detection on surrogate multilayer networks for a given pair of $(\gamma _{r}, \omega )$ values. We then calculate the modularity values of the detected community structures and compute the average modularity, $Q_{surr}$. Next, we perform modularity maximization for $\mathscr {M}$ *c* times and compute the average of the modularity values for the *c* community structures, $Q_{obs}$. This process is repeated for different pairs of $(\gamma _{r}, \omega ) \in \Gamma _{r} \times \Omega$ where $\Gamma _{r}$ and $\Omega$ are given sets of resolution parameters and inter-layer scales, from which the optimal parameter values are searched. The pair with the largest difference, $Q_{obs} - Q_{surr}$, is selected as the optimal parameter values.

### Group community detection

Once the community structures of the multilayer networks for a group of subjects are detected, it is often desirable to find a group community structure, which summarizes the shared communities across subjects^18,61,62^. In this paper, we propose a group community structure detection method based on multiplex graphs. Given *L* subjects, for each subject we maximize the modularity function with the optimal $\gamma _{r}$ and $\omega$ values *c* times to obtain *c* community structures. Since modularity maximization is an NP-hard problem^63^, modularity maximization algorithms yield locally optimal results. By running the algorithm multiple times, one can obtain a collection of informative community structures for each subject. From these community structures, for each subject we construct a co-clustering matrix ${\textbf{A}^{h}}$, ${{h}\in \{1,2,\ldots ,L\}}$ where ${A^{h}_{uv}}$ is the number of times nodes *u* and *v* are in the same community for subject ${{h}}$ across all runs. The resulting *L* co-clustering matrices can be modeled as the layers of a multiplex graph, where each layer is an undirected, weighted graph corresponding to a subject. The group community structure is then found using Spectral Clustering on Multi-Layer graphs (SC-ML)^64^, which finds a common community structure shared by the layers of a multiplex graph. SC-ML applies spectral clustering to a modified Laplacian defined as:

10 $$\begin{aligned} {\textbf{L}_{mod} = \sum _{{{h}}=1}^L \textbf{L}^{h}- \alpha \sum _{{h}=1}^L \textbf{U}^{h}{\textbf{U}^{{h}}}^\top ,} \end{aligned}$$

where ${\textbf{L}^{{h}}}$ is the normalized graph Laplacian for layer ${{h}}$ defined as ${\textbf{L}^{{h}} = (\textbf{D}^{{h}})^{-1/2}(\textbf{D}^{{h}} - \textbf{A}^{{h}})(\textbf{D}^{{h}})^{-1/2}}$, ${\textbf{D}^{{h}}}$ is the diagonal matrix of node strengths and ${\textbf{U}^{{h}}}$ is the low-rank subspace embedding of layer ${{h}}$. In this work, we set $\alpha =0.5$, following the guidelines in^64^. Algorithm 1 gives the complete procedure to obtain group community structure from a given set of multilayer networks.
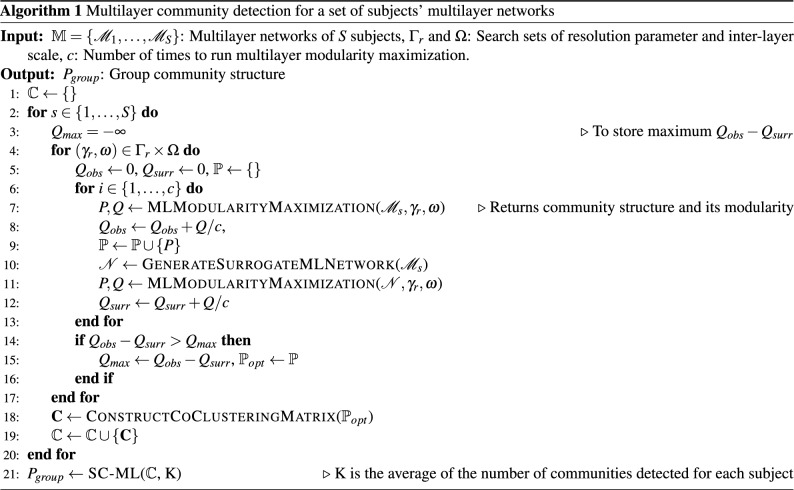


## Results

### Optimal resolution and scale parameters

Using the statistical testing approach described in Materials and Methods, we first study the optimal values of $\gamma _{r}$ and $\omega$. For each subject and each response type, 100 surrogate networks are generated and their community structures are found for each $(\gamma _{r}, \omega ) \in \Gamma _{r} \times \Omega$, where $\Gamma _{r} = \{\gamma _{r} : \gamma _{r} = 0.95 + 0.0025n, n \in \{0, 1, \dots , 40\}\}$ and $\Omega = \{\omega : \omega = 0.0 + 0.0125n, n \in \{0, 1, \dots , 40\}\}$. For each subject, 100 community structures are detected for each $(\gamma _{r}, \omega ) \in \Gamma _{r} \times \Omega$. Modularity values of these community structures are evaluated and the optimal $\gamma _{r}$ and $\omega$ values for each subject are then found from $Q_{obs} - Q_{surr}$.

**Figure 3:**
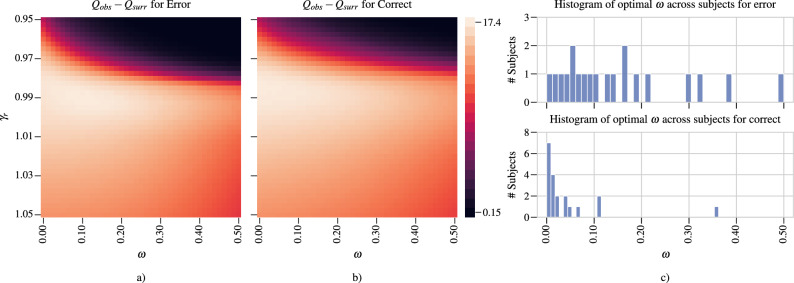
Selection of the resolution ($\gamma _{r}$) and inter-layer scale ($\omega$) parameters: (**a**) and (**b**) show the average of $Q_{obs} - Q_{surr}$ across 20 subjects for error and correct responses, respectively. (**c**) Shows the histogram of optimal $\omega$ values for error (top) and correct (bottom) responses across subjects.

Figure 3a,b show the average of $Q_{obs} - Q_{surr}$ across subjects for error and correct responses, respectively. For both response types, optimal $\gamma _{r}$ is found to be close to 0.99, while optimal $\omega$ values are observed to be more diverse across subjects, ranging between 0.0 and 0.2 for error and between 0.0 and 0.1 for correct. In Fig. 3c, we plotted the histograms of the optimal $\omega$ values across subjects for both response types. This figure shows that the optimal $\omega$ values are non-zero for all subjects except one for the error response. On the other hand, for correct response, the optimal $\omega$ values for 7 subjects is 0, while most of the remaining subjects have optimal $\omega$ values close to 0.

### Consistency of community structures for error and correct

**Figure 4:**
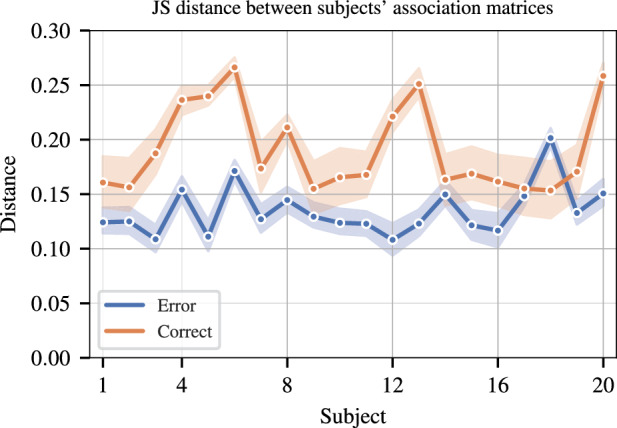
Consistency of the community structure for error and correct responses as measured by JS distance. Average JS distance of each subject with respect to other subjects is shown. Shaded area is the 95% confidence interval.

After obtaining the optimal community structure for each subject and both response types, the consistency of community structures across subjects within each response type is assessed. A multiplex graph is constructed where layer ${{h}}$ corresponds to ${{h}}$th subject’s co-clustering matrix as described in Materials and Methods. The distance between any two layers is used to quantify the consistency of the community structures for those two subjects. Jensen-Shannon (JS) distance for graphs^65^, which is always in [0, 1] and is shown to be effective in assessing similarity of graphs based on their community structure^65^, is used as the distance measure. Figure 4 shows the average JS distance between each subject and the others for each response type. This plot shows that the average distance for each subject with respect to the other subjects is lower for error response compared to the correct response.

### Group community structure for error and correct responses

**Figure 5:**
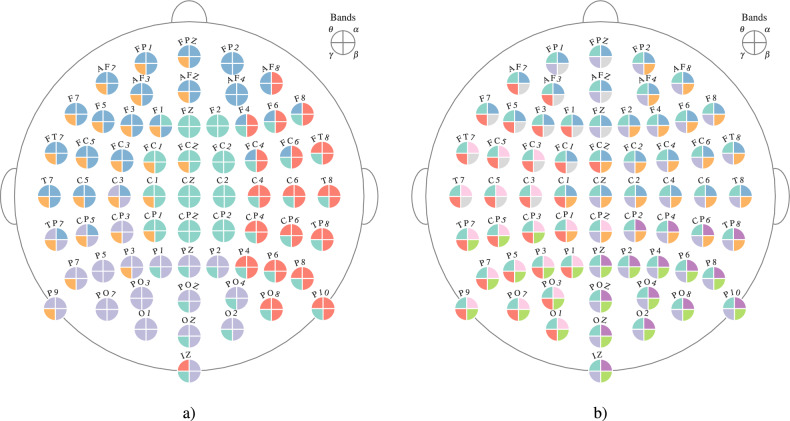
Multilayer group community structures for error (**a**)) and correct (**b**)) responses. Each electrode is shown with a circle with 4 quadrants, corresponding to the 4 frequency bands. Different colors represent different communities. Correspondence of the quadrants to the frequency bands are shown at the upper right corners of (**a**) and (**b**).

Once the optimal community structures are obtained for each subject and for each response type, the group community structure is detected using SC-ML. The number of communities is determined as the average of the number of communities detected for each subject. These values are 5 and 9 for error and correct responses, respectively. Figure 5 illustrates the group community structure for error and correct responses for the multi-frequency networks. For error response, the group community structure consists of communities that include nodes from multiple layers. Community structure of $\theta$, $\alpha$ and $\beta$ are found to be very similar to each other. On the other hand, the community structure for the $\gamma$ is different and has one within layer community, while the rest are across layers. For correct response, all communities are within a single layer. Nodes in the $\theta$ band are all assigned to a single community, while the other bands have distinct community structures.

**Figure 6:**
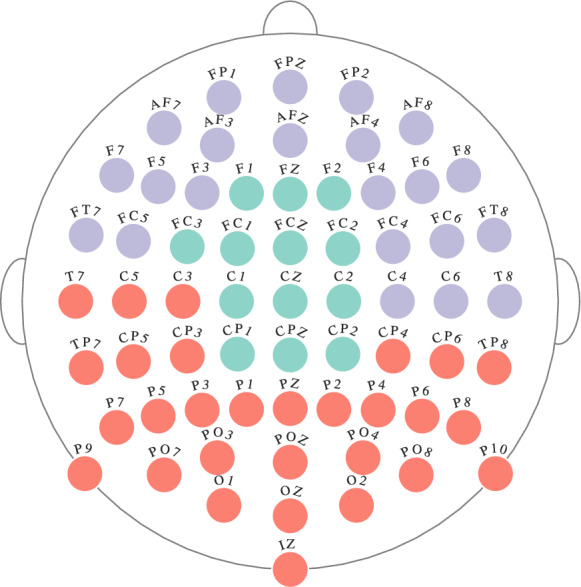
Community structure of $\theta$ band functional connectivity network found by maximizing the single-layer modularity function (see (7)) for error response. Each electrode is shown with a circle where the different colors correspond to different communities.

In order to better interpret the multilayer community structure, community structure for $\theta$ band is detected using single-layer modularity (see (7)). In particular, for each subject the community structure for the $\theta$ band is detected using single-layer modularity for each $\gamma _{r} \in \Gamma _{r} = \{\gamma _{r} : \gamma _{r} = 0.95 + 0.0025n, n \in \{0, 1, \dots , 40\}\}$. The optimal resolution parameter is selected using the surrogate network approach. Using this optimal resolution parameter, group community structure for $\theta$ band for a given response type is found using SC-ML. The number of communities is determined as the average number of communities detected for each subject’s $\theta$ band. Figure 6 shows the group community structure for $\theta$ band for error response. We do not consider the community structure for the correct response in the $\theta$ band, since all of its nodes were assigned to a single community with the proposed multilayer modularity as shown in Fig. 5a. Comparing Fig. 6 with Fig. 5a, it can be seen that there are similarities between the community structures detected by single-layer and multilayer modularity maximization. For instance, the green community in Fig. 6 is also detected in Fig. 5a. Similarly, most of the nodes in purple and red communities in Fig. 6 are in the same communities in the structure detected by the proposed multilayer modularity.

## Discussion

The study of the community structure in multilayer functional connectivity networks reveal some interesting differences between error and correct responses at both the individual and group level. First, we observe the different role that inter-layer coupling plays in community formation for error vs. correct response. At the individual subject level, Fig. 3 illustrates that while inter-layer connections are not important for the community structure of correct response as indicated by the optimal value of the scale parameter, $\omega$, being close to 0 for the majority of subjects, they are influential in community formation following the error response. Our prior work comparing PAC between response types supports this observation as there is significantly higher cross-frequency coupling during error monitoring^42^. This increased cross-frequency coupling is between low frequency cognitive control signals which are activated after an error response and high-frequency oscillations related to motor activity and visual processing in the gamma band^66^.

At the group level, the community structures in Fig. 5, show a community comprised of the frontal-central nodes corresponding to the medial prefrontal cortex (mPFC), e.g. Fz, FCz, FCz, FC2, in the $\theta$ and $\alpha$ bands with parietal-occipital nodes corresponding to the visual , e.g. Pz, POz, Oz, and motor cortices, e.g. C2, C4, C6, in the $\gamma$ band during ERN. mPFC is known to play an important role during ERN. In particular, it is thought to detect conflicts and recruit additional resources from other brain areas including the lateral prefrontal cortices, visual and motor cortices to coordinate task relevant large scale networks and support adaptations of goal-directed behavior^67^. Physiologically, these interactions may occur through local and long range synchronized oscillation dynamics, particularly in the theta range (4-8 Hz). While this mPFC community structure in $\theta$ band has been observed in prior work that indicates the role of mPFC during ERN^47^, the cross-frequency nature of this community is a new finding made possible by the proposed multilayer model. Our recent work shows that the phase of the $\theta$ band oscillations from the frontal-central regions modulate the amplitude of the $\gamma$ band oscillations in the parietal-occipital regions following an error response supporting this finding^68^. Prior studies from others also hypothesize that error-related negativity initiates the medial frontal based top-down control mechanisms to improve the performance after an error response^69^. More recently, it has been proposed that low frequency network oscillations in prefrontal cortex, e.g. theta, guide the expression of motor-related activity in action planning and guide perception-related activity, e.g. gamma, in memory access^70^. Thus, the communities detected are consistent with previous literature reflecting higher theta-gamma coupling in the medial frontal cortex and relating this with error-related negativity. Another observation that can be made from Fig. 5a is that the nodes corresponding to $\alpha$ and $\beta$ bands are primarily in the same communities. This is line with recent work that indicates interlayer connectivity is dominated by one-to-one interactions for alpha-to-beta bands while for $\theta$-$\gamma$ band networks, there are additional interlayer connections between distant nodes in addition to the one-to-one connections^17^. The community structure for the correct response is mostly within-layer indicating the lack of coupling across different frequency bands.

When the group community structure for $\theta$ band in Fig. 6 is compared to the that of Fig. 5a, some similarities are observed. As mentioned before, the community consisting of frontal and central electrodes in Fig. 6 is also found by the proposed multilayer community detection method. Partitioning of the remaining electrodes is also consistent across both Figures. In order to quantify the similarity of community structures of $\theta$ band shown in Figures 5a and 6, we use Normalized Mutual Information (NMI)^71^. For Figs. 5a and 6, NMI is found to be 0.60, indicating an agreement between the community structures in the $\theta$ band detected by single-layer and multilayer modularity maximization methods. This consistency between the community structures across the two definitions of modularity is enabled by the way we define multilayer modularity. Our definition of multilayer modularity takes the heterogeneity of edge strengths into account, thus we are able to resolve the structure within layers.

Finally, Fig. 4 shows that there is more group level consistency in terms of topological organization for the error response compared to the correct response. This is in line with prior work^47^ that shows that the organization of the functional connectivity networks for correct response is similar to pre-stimulus networks. Thus, there is more variation across subjects for the correct response compared to response-evoked networks following an error response.

## Conclusions

This paper introduced a multilayer model of functional connectivity of the brain. In particular, we provided a data-driven approach to construct multi-frequency connectivity networks where layers correspond to different frequency bands. The resulting networks capture both within and cross-frequency coupling in a single framework. We then introduced a new definition of modularity for multilayer networks such that the null model preserves the heterogeneity of edge weights across layers. The community detection algorithm resulting from the maximization of this multilayer modularity function is applied to EEG data collected during error monitoring. The results indicate that following an error response, the brain organizes itself to form cross-frequency communities. This cross-frequency community formation is not observed for the correct response which indicates that the cross-frequency coupling is primarily associated with cognitive control. Moreover, we observed that the community structures detected for the error response were more consistent across subjects compared to the community structures for correct response.

Future work will consider extension of this multilayer model to higher dimensions, e.g. multi-aspect multilayer brain networks such as temporal multi-frequency connectivity networks. Compared to current work where subjects’ community structure is found separately and then combined through group community detection, future work can use multi-aspect multilayer networks constructed from subjects’ multilayer networks. This approach will allow simultaneous detection of communities of subjects similar to^22^. Future work will also consider different null models in the definition of modularity such as the constant Potts model, which is shown to be resolution limit free^72^. Finally, in this work we aimed to find the optimal resolution and inter-layer scale parameter; future work can focus on a multi-scale approach where the aim is to combine community structures from different resolutions and inter-layer scales^73^.

## Data Availability

The datasets generated during and/or analyzed during the current study are available from the corresponding author on reasonable request. The codes can be accessed at https://github.com/SPLab-aviyente/MLModularityForEEG.
